# GABA_A_ Receptor in the Thalamic Specific Relay System Contributes to the Propofol-Induced Somatosensory Cortical Suppression in Rat

**DOI:** 10.1371/journal.pone.0082377

**Published:** 2013-12-04

**Authors:** Yu Zhang, Chaoping Wang, Yi Zhang, Lin Zhang, Tian Yu

**Affiliations:** Department of Anesthesiology, Affiliated Hospital of Zunyi Medical College, Zunyi, Guizhou, China; Imperial College London, Chelsea & Westminster Hospital, United Kingdom

## Abstract

Interaction with the gamma-aminobutyric-acid-type-A (GABA_A_) receptors is recognized as an important component of the mechanism of propofol, a sedative-hypnotic drug commonly used as anesthetic. However the contribution of GABA_A_ receptors to the central nervous system suppression is still not well understood, especially in the thalamocortical network. In the present study, we investigated if intracerebral injection of bicuculline (a GABA_A_ receptor antagonist) into the thalamus ventral posteromedial nucleus (VPM, a thalamus specific relay nuclei that innervated S1 mostly) could reverse propofol-induced cortical suppression, through recording the changes of both spontaneous and somatosensory neural activities in rat’s somatosensory cortex (S1). We found that after injection of bicuculline into VPM, significant increase of neural activities were observed in all bands of local field potentials (total band, 182±6%), while the amplitude of all components in somatosensory evoked potentials were also increased (negative, 121±9% and positive, 124±6%).These data support that the potentiation of GABA_A_ receptor-mediated synaptic inhibition in a thalamic specific relay system seems to play a crucial role in propofol-induced cortical suppression in the somatosensory cortex of rats.

## Introduction

The mechanism by which consciousness is suppressed during anesthesia remains unknown. Many studies have investigated how gamma-aminobutyric-acid-type-A (GABA_A_) receptors are affected by numerous anesthetic substances [1,2]. The prevailing view is that GABA_A_ receptors are the molecular targets most directly related to producing the clinical effects of anesthetics on consciousness and immobility[3]. However, the link between anesthetic actions and loss of consciousness can only ultimately be explained by understanding the functional effects of anesthetics on specific neuronal pathways. 

The thalamus is the major entrance that allows the flow of sensory information from the periphery into the cortex[4]. The thalamocortical system also plays a central role in information integration in the brain [5,6]. The disruption of thalamocortical connectivity leads to a state that essentially isolates the cortex from the environment and is the main reason of loss of consciousness [7]. 

In particular, the thalamus can be divided into two major divisions, the specific relay nuclei and the more diffusely projecting “nonspecific” nuclei. The two parts of thalamus may collaborate to accomplish the integration task [8]. The specific system is responsible for the transmission of sensory information, while the nonspecific system is engaged in the control of cortical arousal and temporal conjunction of information across distributed cortical areas [9]. These considerations emphasize the importance of the nonspecific thalamocortical system in information integration and raise the possibility that its dysfunction may be a primary, and possibly a major mechanism of anesthetic-induced unconsciousness.

It has been shown in our previous work [10] and other functional human brain imaging studies (PET [11] and MRI [12]), that the thalamus is inactivated during general anesthesia at loss of consciousness, suggesting that the thalamus may serve as a consistent regional target of anesthetics (except ketamine). For that reason, it seems likely that at the beginning of general anesthesia, anesthetics impact the thalamocortical pathways in a yet unknown pattern which disturbs the functional interactions within neural networks involving the thalamus and cerebral cortex, thereby causing an instantaneous loss of consciousness.

However, none of these currently available imaging techniques can directly offer neuronal activity data. Rather, they infer changes in activity from changes in blood flow, glucose metabolism or oxygen concentration. Because these surrogate measures might be affected by anaesthetics independent of any changes in neuronal activity (for example, anaesthetics might change the vascular resistance), the interpretation of results has to be tentative. Therefore, the current study was motivated by the fact that very little direct evidence is gained about the role of GABA_A_ receptors in specific system of the thalamocortical pathways during general anesthesia. In order to obtain the direct evidence experimentally, we investigated the changes of local field potentials (LFP) and somatosensory evoked potentials (SEP) in somatosensory cortex (S1) with intracerebral injection of GABA_A_ receptors antagonist bicuculline into a specific thalamus relay nuclei: ventral posteromedial nucleus (VPM), which innervated S1 mostly [13]. All our work addressed the question as to whether GABA_A_ receptors in thalamus specific system contribute to attenuation of the thalamocortical activity during propofol anesthesia.

## Methods

### Animals and ethics statement

Sprague–Dawley rats of either sex, weighing 250 to 350 g, were purchased from the Center of Laboratory Animals in Shanghai Institutes for Biological Sciences (Shanghai, China). All animals were housed on a 12 hr light/dark cycle under constant temperature (22 ± 2°C) and humidity (50%) and had *ad libitum* access to food and water. All studies were performed in accordance with the “Guide for the care and use of laboratory animals” in China (no. 14924, 2001). All animal surgical procedures were approved by the Committees on Investigations Involving Animals in Zunyi Medical College, China. The number of animals used was distributed as follow: Spontaneous local field potential recording (n = 8), and whiskers stimulation evoked somatosensory potential recording (n = 11).

### Surgical procedures

The rats were anaesthetized by intraperitoneal injection of 80 mg/kg of propofol 2% (AstraZeneca S.p.A, Caponago, ITA) and fixed in a stereotaxic frame (Rewardst, Shenzhen, CHN). Propofol 2% was then micro-pumped (0.8-1.5 ml/h) through the caudal vein to maintain anesthesia during the whole experimental procedure. A tracheotomy was performed, and a short length of polyethylene tubing was inserted into the trachea as a cannula. The skull was exposed, and a 5 × 5 mm craniotomy was made to expose the S1 representation area in the right hemisphere (Bregma coordinates: 2.0 mm posterior, 5.5 mm lateral, and 0.8-1.5 mm depth, Paul Halasz & Lewis Tsalis 5th). Another smaller (4 × 3) craniotomy was made for microinjections of VPM (Bregma coordinates: 3.0 mm posterior, 2.7 mm lateral, and 5.8-6.2 mm depth, Paul Halasz & Lewis Tsalis 5th). During the experiments, the animals breathed spontaneously, and the respiratory frequency was monitored every 5 min. Core temperature was kept at 37 ± 0.5°C by a feedback-controlled heating blanket (Institute of Biomedical Engineering, Tianjin, CHN). The depth of anesthesia in rats was maintained at the light anesthesia level with evidence of the lack of voluntary movement, decreased muscle tone and no reaction to painful stimulation but without any cardiorespiratory function being compromised. Experiments were terminated by barbiturate overdose when the physiological conditions of rats could not be maintained with in normal ranges. 

### Neuronal recording and whisker stimulation

Spontaneous local field potentials (LFP) and whiskers-stimulation-evoked somatosensory evoked potentials (SEP) were recorded from the S1 with extracellular tungsten microelectrodes (tip resistance 2 MΩ; WPI, Shanghai, CHN). A stainless steel screw that placed above the skull served as a reference. These microelectrodes were slowly and perpendicularly moved by a hydraulic microdrive (Narishige, Tokyo, Japan) to a depth of 1-1.2 mm below the dura (with impedance of 4-6 MΩ). The principal whisker of the receptive fields was determined by manual movement of the whiskers while slowly lowering the electrodes. The whisker whose displacement elicited the most vigorous response with the shortest latency was identified as the principal whisker. Then this whisker were trimmed to a length of 10 mm and attached to a stimulator probe that gives approximately 3 mm of movement in a caudal direction. The probe attached to a feedback-controlled electromechanical stimulator (Somedic, Stockholm, Sweden) with constant displacement amplitudes (3 mm amplitude, 50 ms duration delivered at 4 s intervals). Whiskers were deflected for 1 s at 10 Hz in a randomly interleaved pattern; trials were separated by 60 s, and 100 trials were presented to each animal. Cortical neurons responding with sustained spike discharges to vibration of whiskers were selected as SEP.

The neuronal activity was filtered, amplified (300 Hz- 5 kHz, gain: 10000 x; Model 3000; A-M Systems, Washington, US) and displayed by an oscilloscope. All data were stored with 25–35 kHz sampling rate on a hard disk using the Alpha-Map Data Acquisition System (Alpha Omega Engineering, Nazareth, Israel) for off-line analyses.

### Experimental procedure and drug administration

**Figure 1:**
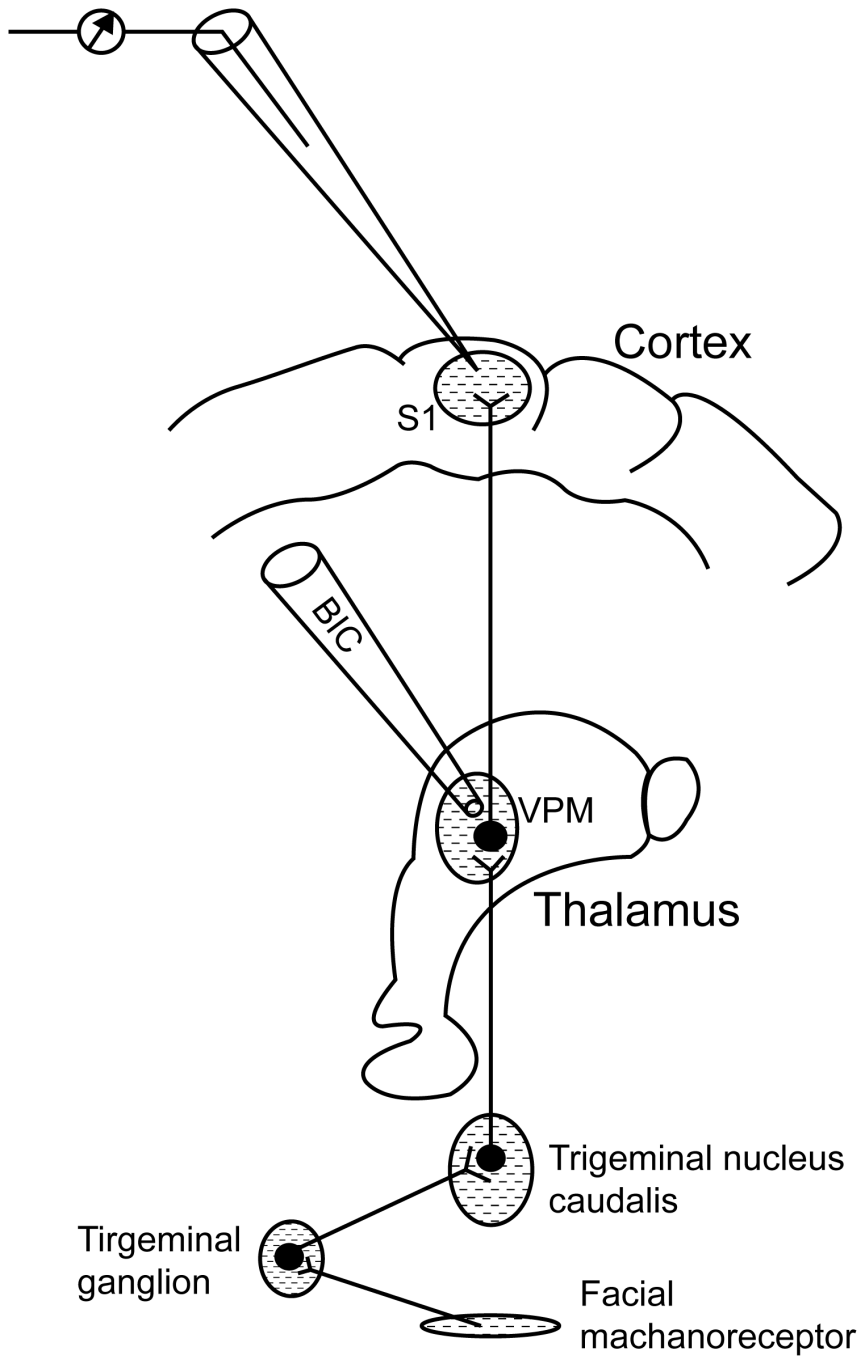
Schematic representation of the somatosensory ascending pathway. The tactile information was transmitted from mechanoreceptors of the facial skin to the primary somatosensory cortex (S1). The tungsten microelectrode allowed extracellular single-unit recordings in S1, while a glass electrode performed the injection of bicuculline (BIC) in the thalamic ventral posteromedial nucleus (VPM).

In order to explore the specific role of GABA_A_-receptor in the thalamocortical pathway under propofol anesthesia, LFP and SEP were recorded with microinjections of the GABA_A_-receptor antagonist bicuculline (BIC) in VPM (Figure 1). Bicuculline methiodide (Sigma, Missouri, USA) was dissolved in 0.9% NaCl saline at concentration of 100 ng/µl. BIC or saline was injected via a glass electrode (tip: 0.5 µm, WPI, Shanghai, CHN) which was connected by a catheter to a microsyringe (10 µl airtight, WPI, Shanghai, CHN). In order to prove the injection process did not change the neural activity of the primary somatosensory cortex, LFP and SEP recording with saline injection in the VPM were performed in 3 extra rats. Injections were performed over a period of 2 min at a rate of 0.1 µl/min in VPM and none of these injections induced physiological change (such as breath rate and heart rate). LFP and SEP recording were performed before and after injection (every 5 min for 20 min). The injection electrode was left in place for 1min after each injection before removal. During the whole recording and injection process, the anesthesia depths of all experimental animals were kept in the middle level (loss of corneal reflex, but maintain purposeful extremity movement to toes clamping with an alligator clip, breath rate of 60-80 min^-1^ and heart rate of 320-340 min^-1^) .

### Histology

**Figure 2:**
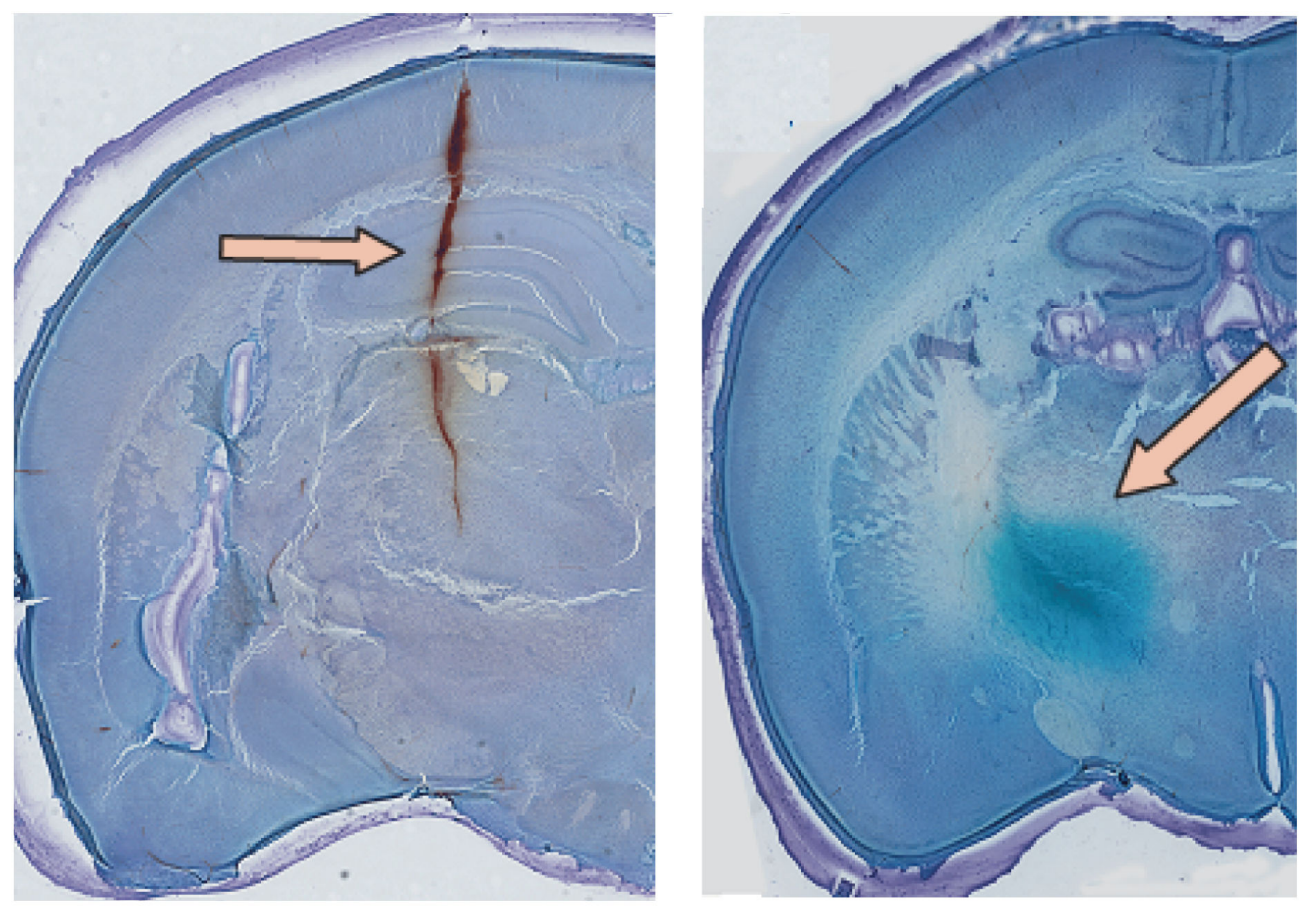
Needle track and diffusion area. Left, the trail of injection electrode (pink arrow, brown track). Right, the area of BIC diffusion in VPM (pink arrow, blue halo).

At the end of testing, the animals were killed with an overdose of pentobarbital (Pentoject; Animalcare, York, UK). Chicago Blue (Sigma, Missouri, USA) was injected via the same injection electrode that placed in VPM (Bregma coordinates: 3.0 mm posterior, 2.7 mm lateral, and 5.8-6.2 mm depth) during drug administration. Then the brains were removed and fixed in 4% paraformaldehyde (Sigma, Missouri, USA) in 0.01 M phosphate buffered saline. 80-μm-thick coronal slices containing evidence of the needle tracks were manufactured by a vibroslicer (HM 650V, Thermo, Michigan, USA) (Figure 2, left) and stained with Benzenesulfonic acid (Sigma, Missouri, USA). Chicago Blue Staining allowed the location of the drug administration point to be identified. The stained point was taken as the center of the injection site (Figure 2, right) and marked on coronal sections modified from the atlas of Paul Halasz & Lewis Tsalis 5th. Animals in which the injection sites were not located in the structures of interest were eliminated from data analysis. 

### Data analysis

Offline analysis of the neurophysiologic data were operated with MATLAB (MathWorks, Inc., Natick, MA, USA), Spike2 (CED, Cambridge, UK) and OriginPro 7.5 (OriginLab Corporation, Northampton, MA, USA). The original LFP signals were filtered into 1–60 Hz (total band), 1-4 Hz (delta band),4–8 Hz (theta band), 8–12 Hz (alpha band), 12–25 Hz (beta band), and 25–60 Hz (gamma band). Comparisons of power values were made within each band before and after BIC microinjection. SEP data were band-pass filtered between 1 and 250 Hz. Temporal data segments from SEP were averaged into a high-frequency response, obtained from averaging the final six responses to the 2-Hz stimulus. Previously published results [14] indicated that four response components would be observable in SEP: composed of two negative and two positive waveforms. For each component, the maximum amplitudes were obtained. SEP amplitudes were calculated from the amplitude of the previous waveform. Amplitude represents the vertical distance between adjacent components. Maximum amplitudes in the SEP data were obtained within the following time windows: 0-20, 10-25, 20-50, and 40-100 ms. Power values and amplitude differences seen before and after treatment were compared statistically by paired, two-tailed Student’s t-tests. Statistical significance was accepted at the p < 0.05 level.

## Results

### BIC injection in VPM reverse the propofol induced neural activity inhibition in the primary somatosensory cortex

**Figure 3:**
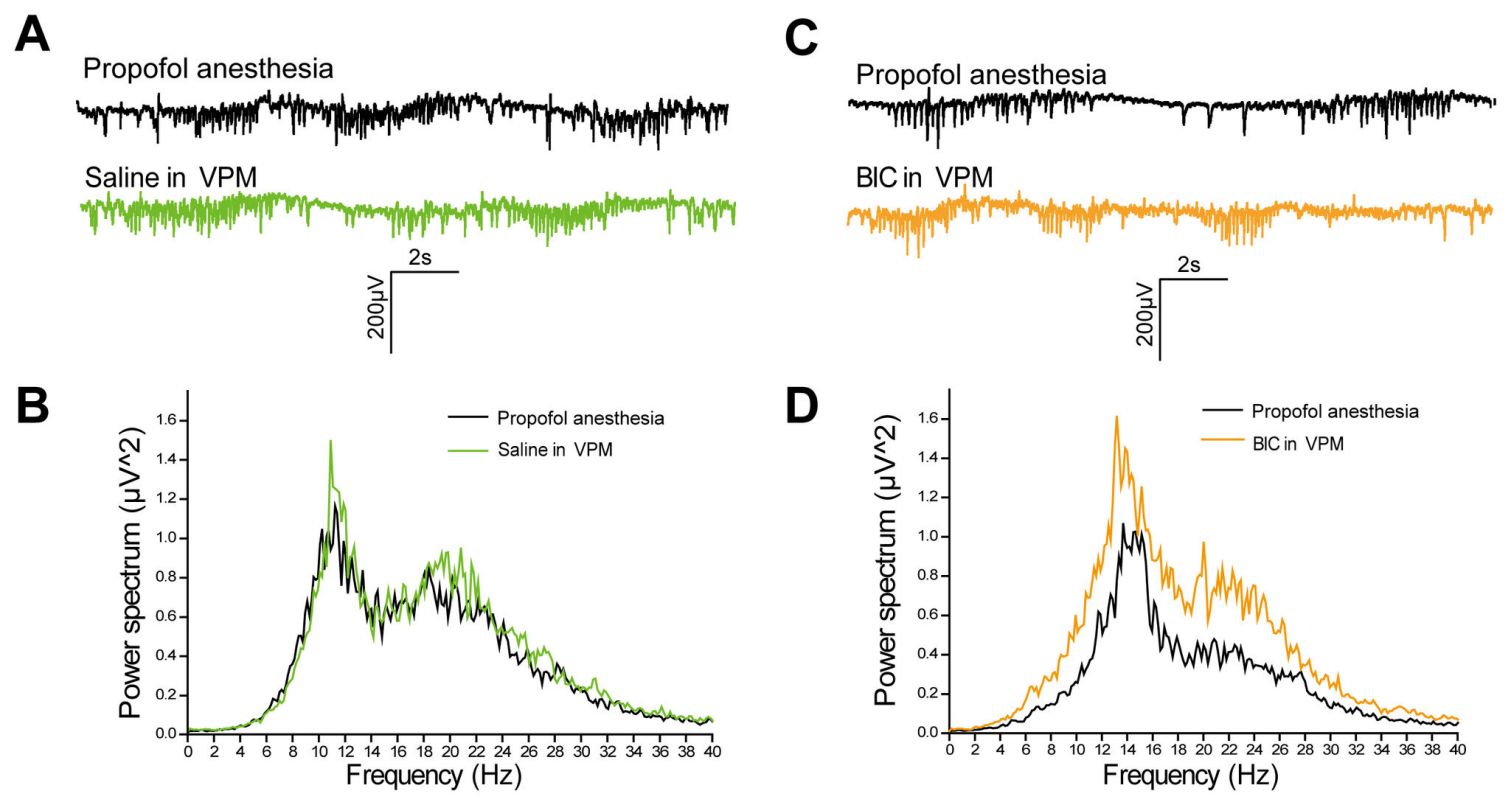
LFP recordings in S1 and power spectral analysis. A, representative section of propofol-induced LFP traces in S1 before and after saline injection in VPM (n=3). B, corresponding power spectral density calculated from A. C, representative section of propofol-induced LFP traces in S1 before and after BIC injection in VPM (n=8). D, corresponding power spectral density calculated from C.

Representative traces obtained from recordings of LFP in the primary somatosensory cortex (S1) of a rat in the propofol anaesthesia state were presented in Figure 3 (A and C; upper panel). Spike discharges representing the LFP were recorded simultaneously. At middle levels of propofol anaesthesia, the LFP consisted of large-amplitude, low-frequency spike-like activity separated by suppression of small-amplitude, high-frequency activity. The subsequent processing of the LFP signals produced power spectrums (Figure 3, B and D). Saline injection in VPM did not change the power spectral density of LFP. In comparison with spectrums before BIC infusions, significant changes in the oscillation power were induced in the frequency ranges of 8-30 Hz (alpha to low gamma bands).The results of power spectrum apparently indicate that BIC injection in VPM reversed the propofol-induced suppression of LFP. 

**Figure 4:**
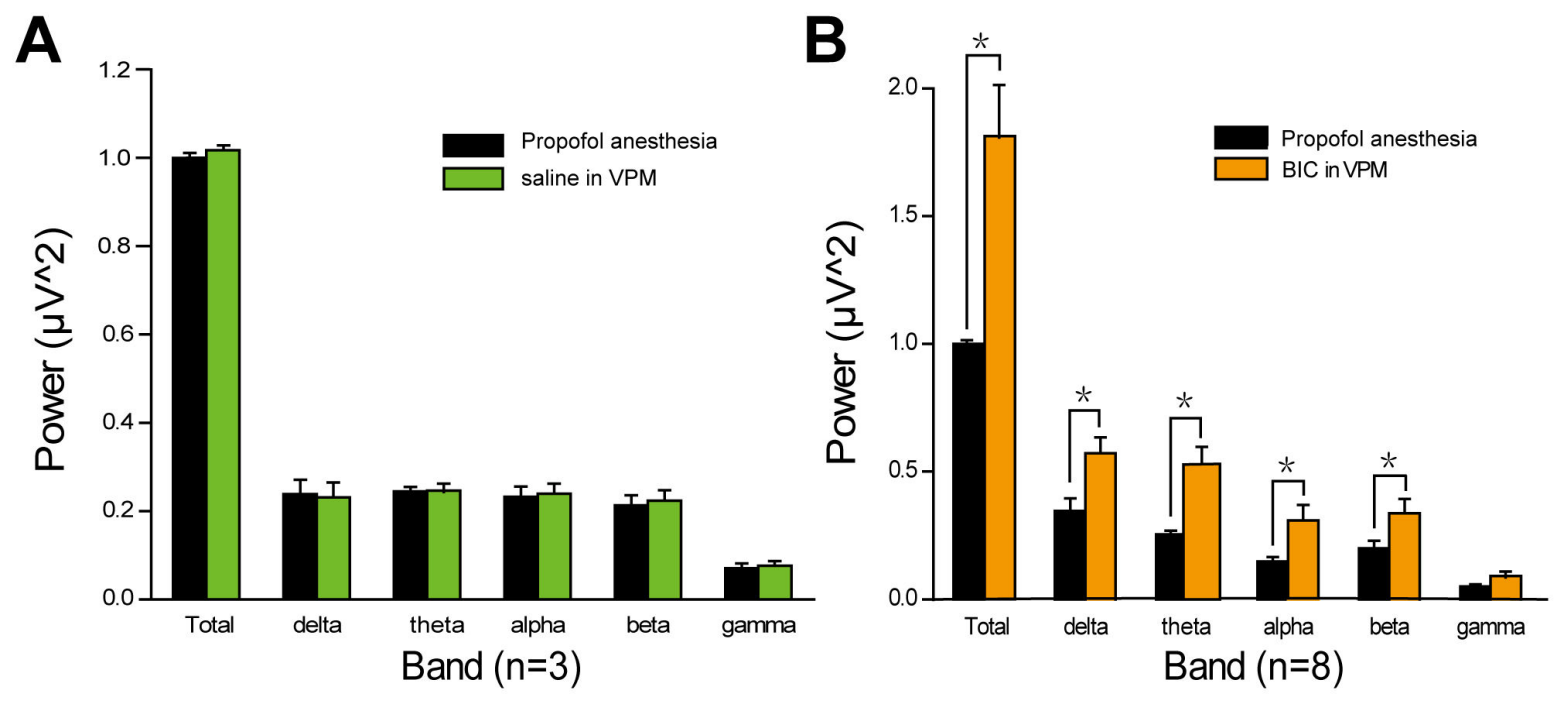
Basal LFP relative power in six frequency bands (total: 1–60 Hz; delta: 1–4 Hz; theta: 4–8 Hz; alpha: 8–12 Hz; beta: 12–25 Hz; gamma: 25–60 Hz). A, saline injection in VPM did not change the values of LFP power in all bands. B, significant rise of power were found in all bands after BIC injection in VPM. Data shown as mean ± S.E.M. (n = 8). *, p < 0.05 versus control.

The basal LFP relative power in five frequency bands was compared between saline injection and BIC injection. The LFP measured in the S1 is dominated with low-frequency components. Saline injection in the VPM did not change the values of power in all bands (Figure 4A), while BIC injection in VPM led to a significant increase relative to that in the control in all LFP bands (Figure 4B). Except gamma band (142±6%, P=0.071), significant trends toward an increase of normalized relative power were observed in all bands in BIC injection in VPM group (total band, 182±6%, P=0.034; delta band, 167±6%, P=0.045; theta band, 198±6%, P=0.039; alpha band, 199±6%, P=0.042; beta bands, 172±6%, P=0.040).The largest increase was observed in theta band and alpha band. There is more relative change in the LFP power in the low (

< 10 Hz)-frequency range with the fact that these frequencies contain nearly all of the total power in the LFP

. 

### BIC injection in VPM boost the somesthetic signal transmission under propofol anesthesia

**Figure 5:**
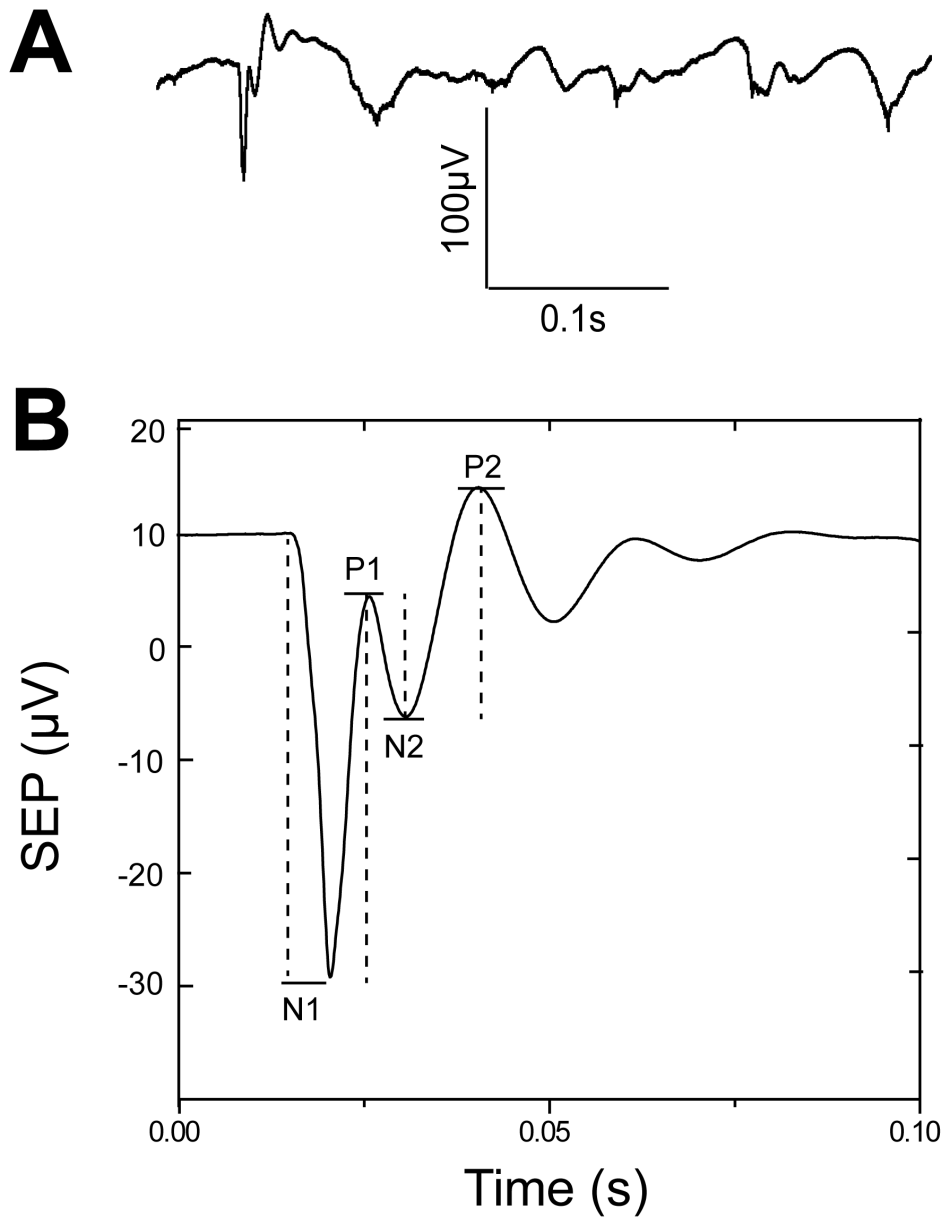
Four response components in SEP. A, Time course plots of SEP responses to stimulus frequencies of 2 Hz during middle propofol anaesthesia. B, Typical SEP response with four components (N1, P1, N2, P2). Dashed lines show how the amplitude of each was calculated.

The origins of average SEP responses, in response to whisker deflection, elicited from a single animal under middle propofol anaesthesia are shown in Figure 5A, which also provides time courses of the SEP response. The S1 area SEP consisted of four peaks (Figure 5B) began with early negative (N1) and positive (P1) sharp waves, followed by slower negative (N2) and positive (P2) waves. There were distinct spatial of the topographical distributions of the four peaks, suggesting that they were generated by different but overlapping neuronal subpopulations. The amplitudes to be calculated for each response component were illustrated in Figure 5B. 

**Figure 6:**
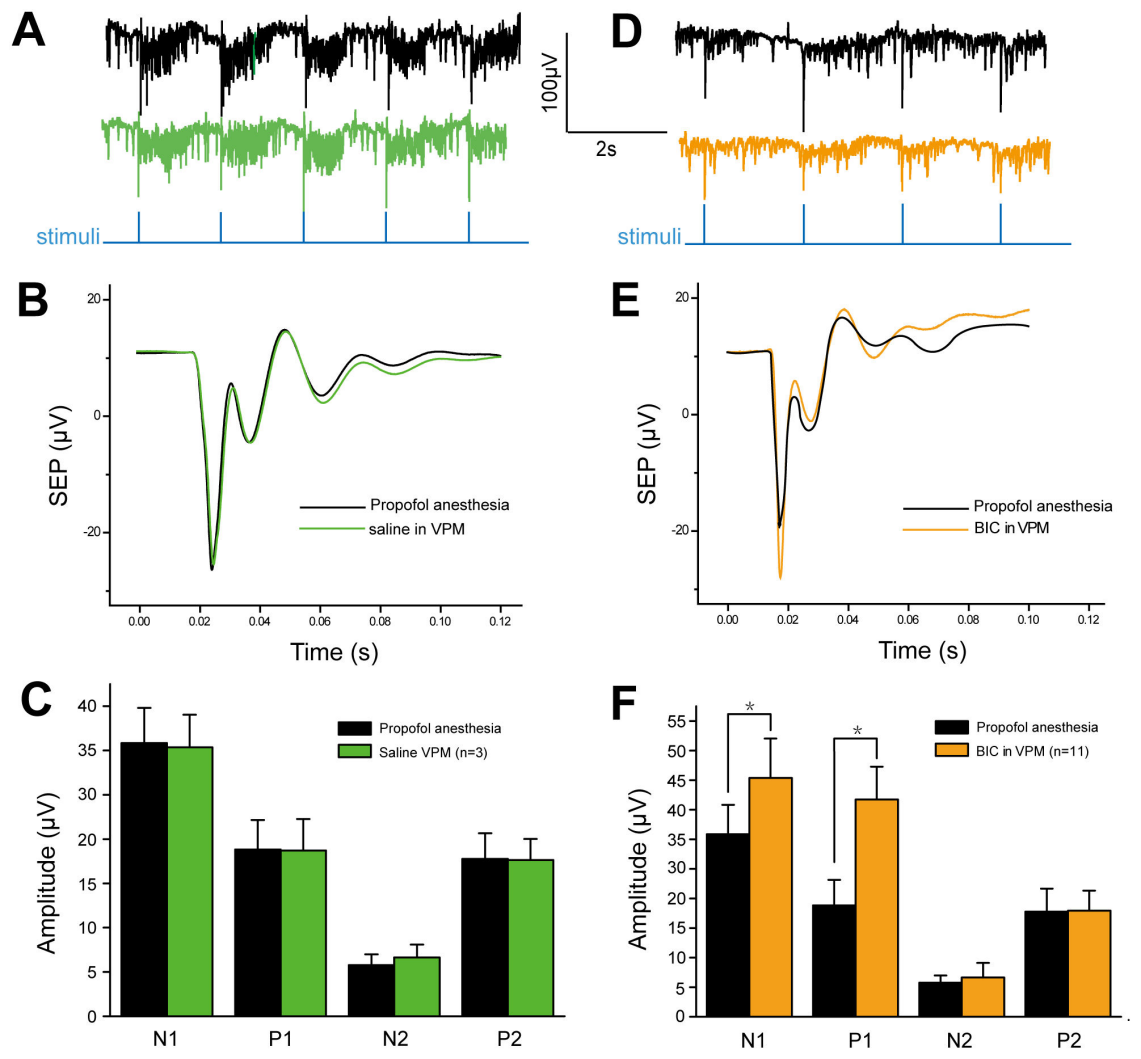
SEP responses before and after injection of saline/BIC in VPM. A and D, original traces. B, time course plots of SEP responses before and after saline injection in VPM (n=3). C, amplitudes of four components of SEP calculated from B, saline injection in VPM did not change the values of all components. C, SEP responses before and after BIC injection in VPM (n=11). D, increase in all response components were observed after BIC injection in VPM. Data shown as mean ± S.E.M. (n = 11). *, p < 0.05 versus control.

Original traces of SEP responses before and after injection of saline or BIC in VPM are shown in Figure 6 (A and D). Time course plots of SEP responses before and after saline or BIC injection in VPM are shown in Figure 6 (B and E), from which amplitude data have been extracted and been shown in Figure 6 (C and F). Saline injection in VPM did not change the amplitude of all components, while the most noticeable effect was BIC injection in VPM caused a raise in amplitude of all SEP response components: both negative and positive response components were increased, on average, by 121±9% and 124±6% as compared with the response before BIC injection. The average amplitudes of all components of SEP responses to 2-Hz stimulation were increased by 123±9% after BIC injection in VPM, whereas the N2 and P2 component lacked significant difference. Changes in SEP response latencies (for any response component) were not as widespread as was observed for changes in amplitude.

### BIC injection in S1 can not reverse the propofol induced inhibition of LFP and SEP

**Figure 7:**
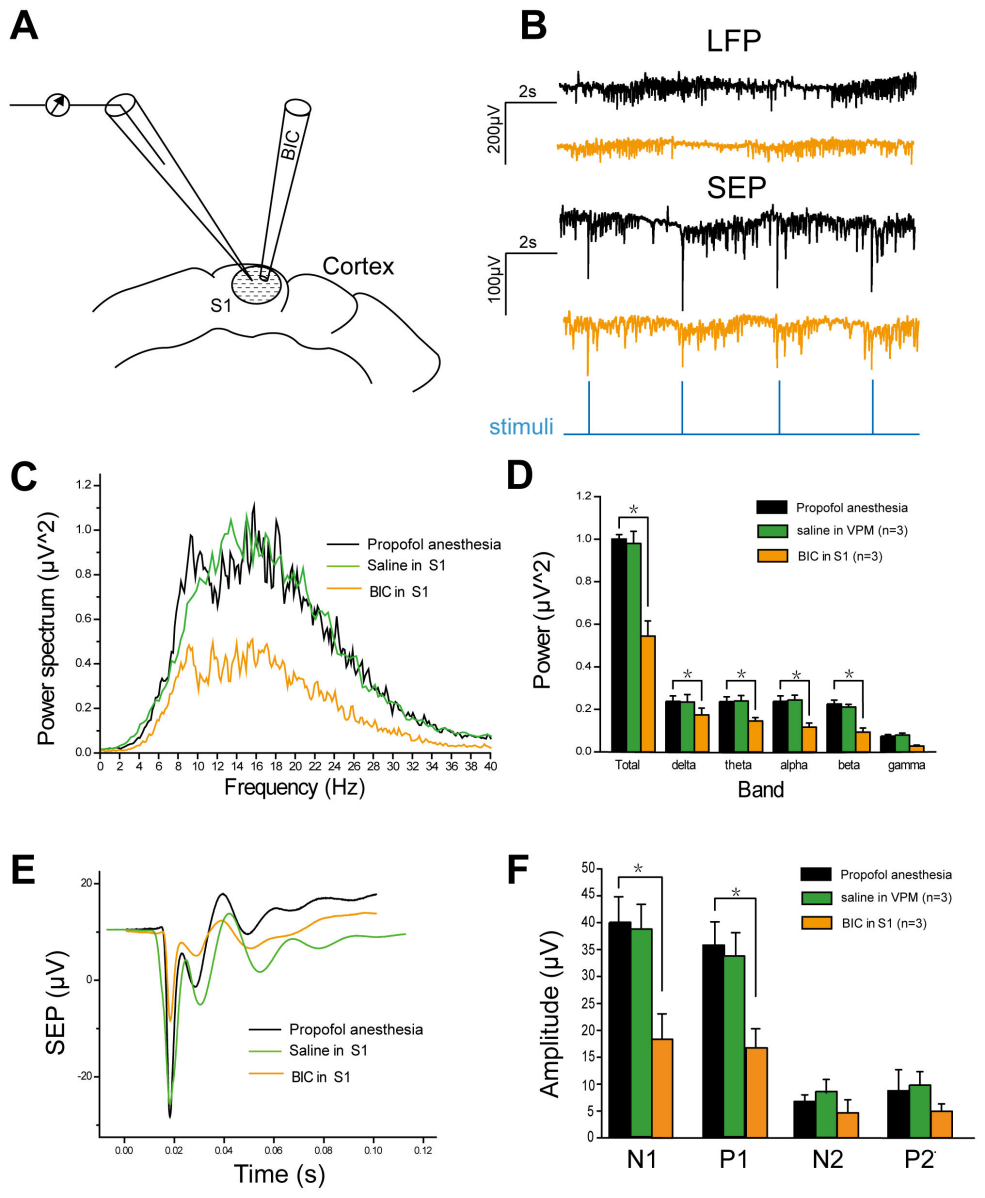
Results of LFP and SEP with BIC injection in S1. A, Schematic representation of the altered injection site (in S1 area) of BIC. B, original traces of LFP and SEP traces before and after BIC (and saline) injection in S1. C, power spectral density of LFP. D, basal LFP relative power in six frequency bands. E, time course plots of SEP responses before and after BIC (and saline) injection in S1. F, amplitudes of four components of SEP calculated from E. Data shown as mean ± S.E.M. (n = 5). *, p < 0.05 versus control.

We next asked whether local microinjection of GABA_A_ receptors antagonist in somatosensory cortex (S1) could also significantly antagonize the inhibition effect of propofol on S1 cortical neuron. Again, both LFP and SEP recordings were performed in the presence of BIC injection in somatosensory cortex (S1) (Figure 7A). We examined three rats with BIC injection in S1, while saline injection in S1 did not change the value of both LFP and SEP (Figure 7 D and F). To our surprise, the results indicated that, in comparison with BIC injection in VPM, intracerebral injection of BIC in S1 produced a significant decrease of normalized relative power of LFP. The decreases were observed in all bands (total band, 53±5%, P=0.033; delta band, 70±4%, P=0.042; theta band, 63±5%, P=0.037; alpha band, 42±7%, P=0.032; beta bands, 39±5%, P=0.041; gama bands, 49±6%, P=0.067) (Figure 7, C and D). Furthermore, BIC injection in S1 also caused a significant decline of amplitude of all SEP response components (Figure 7, E and F). The N1 response component was decreased by 46±6% as compared with the response before BIC injection, while the P1 component was decreased by 47±8%. The average amplitudes of all components of SEP responses were decreased by 59±7% (Figure 7F).

## Discussion

The most striking and consistent change observed in primary somatosensory cortex, was a increase in neural activities after BIC injection in the VPM. For LFP, BIC injection in VPM caused an increase in relative power in all bands, but the degree of these power variations was more marked in theta to alpha band. For SEP, the growths in all response components were observed in rats with BIC injection in VPM. However, the changes of response latencies were not as robust as the changes in amplitude. 

The level of anesthesia in this study was monitored at all times using various physiological signs. Previous results [15] have indicated that the neural activities of relay neurons in the thalamic VPM nucleus of rats decreases in proportion to the middle depth of anesthesia (the spontaneous activity in VPM neurons ranged from 0.5 to 10 Hz, representing in - 87% of the neurons isolated). Thus the level of anesthesia we have adopted offered a steady recording condition with propofol-inhibited neural activities. 

### The role of GABA_A_ receptors in the specific relay nuclei to propofol anesthesia

In almost all human neuroimaging studies, thalamic depression is a common feature of both inhaled and intravenous anesthesia [16]. It has also been suggested that the thalamocortical system plays a central role in information integration in the brain [5]. Previous studies implied a critical involvement of the nonspecific thalamic nuclei (which engage in the control of cortical arousal and temporal conjunction of information across distributed cortical areas) in the loss and recovery of consciousness in anesthetized animals [17]. However, in particular, the other major division of the thalamus, the specific relay nuclei (which is responsible for the transmission and encoding of sensory and motor information) may collaborate with nonspecific nuclei to accomplish this integration task [8,18].

As described in the preceding text, the thalamic ventral posterior medial (VPM) nucleus is the principal whisker-related thalamic relay nucleus, one of the most classical specific relay nuclei that have been fully studied. The results of our investigation clearly illustrated that infusion of antagonist BIC blocked the GABA_A_ receptor in the VPM, and reversed the effects of propofol in rat’s cortical neural activities (both LFP and SEP). This suggests that the potentiation of GABAergic inhibition in thalamic specific relay nuclei may in fact play an important role in anesthetic action of propofol.

**Figure 8:**
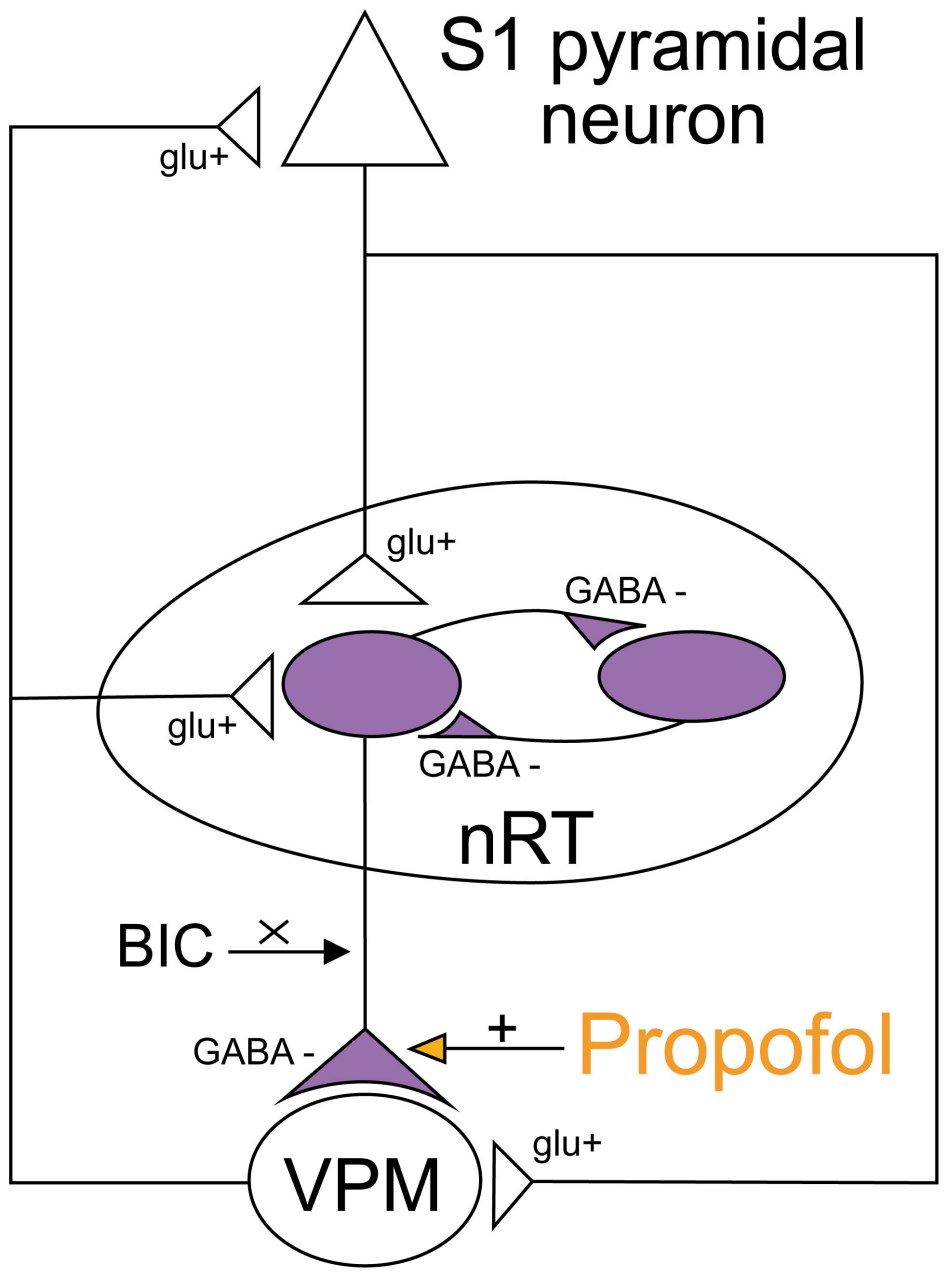
Simplified diagram of the corticothalamic loop comprised of somatosensory cortex (S1), ventral posteromedial relay nucleus (VPM) and the thalamic reticular nucleus (TRN). (+) and (-) correspond to glutamatergic (glu) excitatory and GABAergic (GABA) inhibitory projections, respectively. Propofol may increase the GABA_A_-receptor-mediated synaptic inhibition and thus interrupting the thalamocortical transmission.

VPM neurons receive innervations from the thalamic reticular nucleus (TRN), which consists of GABAergic circuits that cover most of the rostral, lateral and ventral parts of the thalamus (Figure 8) [19]. The TRN inhibitory GABAergic cells and their interconnected networks are particularly well suited for the generation of spindle oscillations (7–14 Hz) that characteristically appear during early stages of sleep and anesthesia [20]. Lee and his colleagues [21] proposed two unique roles for the GABAergic inhibition in rat VPM arising primarily from the TRN. The fast inhibition (GABA_A_-receptor-mediated) can modulate the strength of sensory information without altering the receptive field characteristics of the sensory input, such as size and directional information. To a lesser extent, the late inhibition (GABA_B_-receptor-mediated) regulates the efficacy of sensory input, and perhaps can integrate the inputs from a larger sensory area. As illustrated in Figure 8, the GABAergic TRN neurons loop with glutamatergic cells from VPM and cortex. When the brain is in a wakeful, activated state, the excitatory glutamatergic pathway provides a tonic depolarization of the VPM neurons, tending to prevent them from entering synchronized, oscillatory states, which shall close the “gate” of tactile information procession. The results of present study lead to the notion that propofol could act, at least in part, by boosting GABA_A_-receptor-mediated synaptic transmission and inhibiting these glutamatergic pathways, and thus interrupting the thalamocortical transmission (Figure 8). 

### The effect of propofol on neural reactivity of the primary sensory cortices

Present work has shown a degree of conflicting results. Most previous studies [3,22] indicated that in the sedation level of anesthesia, the neural reactivity of the primary sensory cortices to external stimuli is preserved. Liu and colleagues [23] utilized functional magnetic resonance imaging-guided connectivity analysis to assess the integrity of functional interactions within and between different levels of the task-defined brain regions, and examined how cognitive networks involved in auditory verbal memory are maintained in wakefulness, disrupted in propofol-induced deep sedation, and re-established in recovery. They found that task-related responses persisted in the primary auditory cortex, but vanished in the inferior frontal gyrus and premotor areas (representing high-order processing of the memory task) in deep sedation. Upon such results, they suggest that propofol disrupts cognition by blocking the projection of sensory information to high-order processing networks and thus preventing information integration. Gilles et al [24] also conclude that, based on their results of blood oxygenation level-dependent functional magnetic resonance imaging, the primary and association auditory cortices remain responsive to complex auditory stimuli during anesthesia. Experiment of visual sensory cortices [25] also found that inhalational anesthetics preferentially impair frontal-posterior information transfer at high gamma frequencies, but do not change the event-related potentials in the primary visual cortex. Their findings are consistent with a differential effect of anesthetics on the different functional partitions of the thalamocortical system. In a word, the specific thalamic pathways that encodes and relays sensory information is essentially preserved in anesthesia, whereas the nonspecific thalamic system that facilitates temporal integration of information is consistently disrupted during suppressed consciousness. 

However, there are studies offering support to our results, such as Pierre et al [26] reported that propofol-induced decrease in consciousness linearly correlates with decreased corticocortical and thalamocortical connectivity in frontoparietal networks, and a negative correlation was identified between thalamic and cortical activity in these networks during induced unconsciousness. Alkire [27] also suggested that they found impairment of thalamocortical and corticocortical projections during the general-anesthetic-induced unconscious state.

The lack of consensus may be related to the differential effects of propofol on the specific and nonspecific thalamic networks. In the current study, only one specific thalamic nucleus (VPM) was chosen because they admitted a relatively clear identification from the anatomical images. Most specific nuclei were lumped together with no further differentiation into sensory, motor, and other nuclei. Higher-order thalamic relay nuclei may also mediate corticocortical communication innervating primary and higher-order sensory cortices [28]. Thus, a more refined differentiation of thalamocortical functional connectivity may bring further insight. 

### Analysis of the inconsistent results from local microinjection of BIC in S1

The results indicated that the local microinjection of competitive GABA_A_ receptor antagonist in S1 was unlikely to antagonize the effect of propofol on cortical neuron activity. This suggests that, in S1 area, propofol may not exert its inhibition action by potentiating GABA at the GABA_A_ receptor.

Appropriate explanation can be found form pervious *in vitro* experiments which also indicated that other targets may also be important in the cortical effects of propofol: Such as ZD-7288, a HCN channel blocker (HCN channels represent the molecular basis for native hyperpolarization-activated cationic current [29]), can antagonize the propofol induced membrane hyperpolarization and suppression of action potential discharge in cortical neurons [30]; BIC were also found unlikely to antagonize the effect of propofol on neural activity on an *in vitro* model of epilepsy. However, a specific chloride channels blocker, picrotoxin, partly antagonized the effect of propofol on neural activity [31]. Based on these findings, the researchers suggested that propofol exerts its anticonvulsant action by promoting inhibitory synaptic transmission directly on the chloride channel, not by potentiating GABA at the GABA_A_ receptor. 

All together, these pervious works and our results indicated that the overall actions of propofol may involve modulation of ion channels in addition to GABA_A_ receptors, which are consistent with accumulating evidence that general anesthetics do not simply provide widespread suppression of neuronal excitability by boosting GABA_A_ receptor.

## Conclusion

Although we demonstrated the crucial role of GABA_A_ receptors on the propofol-induced suppression of specific thalamocortical functional connectivity, the degrees of GABAergic reduction in the whisker-related thalamic relay system cannot be determined from the current study. A dose-dependent study would be required to obtain this information.

In summary, we demonstrated that infusion of antagonist of GABA_A_ receptor into the VPM significantly reversed the propofol-induced suppression of cortical activity in S1. The results are consistent with the presumed roles of the two thalamic divisions in information and integration as necessary conditions for consciousness. Therefore, we conclude from our results that the potentiation of GABA_A_-receptor-mediated synaptic inhibition in thalamic specific relay system seems to play a crucial role in propofol-induced cortical suppression in rat’s somatosensory cortex.
